# Formation of printable granular and colloidal chains through capillary effects and dielectrophoresis

**DOI:** 10.1038/ncomms15255

**Published:** 2017-05-12

**Authors:** Zbigniew Rozynek, Ming Han, Filip Dutka, Piotr Garstecki, Arkadiusz Józefczak, Erik Luijten

**Affiliations:** 1 Institute of Physical Chemistry, Polish Academy of Sciences, Kasprzaka 44/52, 01-224 Warsaw, Poland; 2 Faculty of Physics, Adam Mickiewicz University, Umultowska 85, 61-614 Poznań, Poland; 3 Applied Physics Graduate Program, Northwestern University, Evanston, Illinois 60208, USA; 4 Institute of Theoretical Physics, Faculty of Physics, University of Warsaw, Pasteura 5, 02-093 Warsaw, Poland; 5 Departments of Materials Science & Engineering, Engineering Sciences & Applied Mathematics, Physics & Astronomy, Northwestern University, Evanston, Illinois 60208, USA

## Abstract

One-dimensional conductive particle assembly holds promise for a variety of practical applications, in particular for a new generation of electronic devices. However, synthesis of such chains with programmable shapes outside a liquid environment has proven difficult. Here we report a route to simply ‘pull' flexible granular and colloidal chains out of a dispersion by combining field-directed assembly and capillary effects. These chains are automatically stabilized by liquid bridges formed between adjacent particles, without the need for continuous energy input or special particle functionalization. They can further be deposited onto any surface and form desired conductive patterns, potentially applicable to the manufacturing of simple electronic circuits. Various aspects of our route, including the role of particle size and the voltages needed, are studied in detail. Looking towards practical applications, we also present the possibility of two-dimensional writing, rapid solidification of chains and methods to scale up chain production.

## Abstract (web summary)

Conductive colloidal chains are promising for electronics but difficult to synthesize outside of a liquid environment. Here, the authors use field-directed assembly and capillary effects to pull conductive particle chains out of a suspension, which remain held together by flexible liquid bridges even after the external field is turned off.

Fabrication of one-dimensional (1D) granular and colloidal materials is of considerable interest, as they offer opportunities for a variety of electronic applications, including granular conductors^1^, flexible electronics for wearable devices^2^ and electromagnetic energy transport^3^,^4^. These 1D patterns can be assembled either from particle groups^5^,^6^,^7^,^8^ or from individual particles^9^,^10^,^11^,^12^,^13^. The latter can also be employed in mechanical contexts, for example, as flexible artificial flagella or cilia^14^,^15^,^16^. Compared to other methodologies such as lithography^2^,^17^, cluster-assisted assembly^18^ and colloidal polymerization^19^, field-directed assembly in electro- or magneto-rheological fluids^20^,^21^ provides a simple, efficient^22^ and controllable approach for particle chain formation. However, it suffers from two major limitations that hinder its application, for example, in electronic-device manufacturing. First, the assembly typically takes place in bulk liquid, which limits the control over positioning of the chains. Second, maintaining the formed structures normally requires a continuous energy supply; once the external field is turned off, the structures disintegrate. Avoidance of this limitation requires special functionalization of the particle surfaces, such as grafting of DNA^23^,^24^,^25^ or polymeric crosslinkers^22^,^26^,^27^ and designing charge groups^10^.

Here we propose a route to overcome both of these limitations, making it possible to easily fabricate self-sustained, printable granular and colloidal chains outside of a dispersion. The central idea is to combine field-directed assembly with capillary effects, as illustrated in Fig. 1. Conductive particles of radius *a* are initially dispersed in a leaky dielectric liquid that only gives rise to weak screening effects, for example, natural or synthetic oil. To initiate chain formation, we bring a needle-shaped electrode to the liquid surface, and apply an electric field through it. The external field polarizes nearby conductive particles. Through dielectrophoresis (DEP), these conductive particles are attracted along the field gradient, that is, towards the tip of the electrode. When the electrode rises, it pulls the particles out of the liquid to form a particle chain. Along the chain, any two adjacent particles are connected by a sphere–sphere liquid bridge. Interestingly, the combined capillary effects of these bridges make it possible to maintain a stable particle chain even after the external field is turned off.

## Results

### Force analysis

The viability of this approach can be estimated from direct force calculations. Stability of the particle chain in the absence of an external electric field requires the capillary force *F*_ss_≈2π*γa* (*γ* the surface tension of the liquid) to exceed the gravitational force acting on an entire chain *F*_g_=4π*Nρga*^3^/3 (*N* the number of particles, *ρ* the particle density and *g* the gravitational acceleration). For the shortest chain (two particles) an upper limit to the particle size is *a*_max_=(3*γ*/2*ρg*)^1/2^. Given *γ*_oil_∼0.01 N m^–1^, *ρ*∼1 g cm^–3^ and *g*∼10 m s^–2^, we estimate *a*_max_∼1 mm. For particles with radii up to several microns, their weight is negligible, as *F*_g_=(*a*/*a*_max_)^2^*F*_ss_≤10^−4^ *F*_ss_, and thus a long chain composed of hundreds or thousands of particles is stable in the absence of an electric field.

To achieve a chain formation by sequentially pulling each individual micron-sized particle out of the liquid, the only requirement is that the DEP force *F*_e_ exerted on it must overcome the downward capillary force *F*_sp_, such that the sphere–plane liquid bridge can be extended and broken. The sphere directly above it is connected to the electrode and stays at voltage *U* with respect to the grounded container. Thus, it carries a charge *Q*=4π*ɛ*_oil_*ɛ*_ο_*aU* (*ɛ*_oil_ the relative permittivity of the medium and *ɛ*_ο_ the vacuum permittivity) and thus generates a radially decaying electric field *E*=*Q*/(4π*ɛ*_oil_*ɛ*_ο_*r*^2^)=*aU*/*r*^2^. Given the conventional formula^28^ for the DEP force *F*_e_=2π*ɛ*_oil_*ɛ*_ο_*a*^3^*κ*∇(*E*^2^), its action on the sphere beneath it is *F*_e_=8π*ɛ*_oil_*ɛ*_ο_*a*^5^*κU*^2^/*d*^5^, where *d* is the centre-to-centre distance between the two spheres, and *κ* the Clausius–Mossotti factor, which approaches unity for highly conductive particles. In the limit that the two spheres are at contact, *d*→2*a*, we obtain *F*_e_=π*ɛ*_oil_*ɛ*_ο_*κU*^2^/4. Thus, the applied voltage *U* should be at least *U*_min_*=*(4*F*_sp_/π*ɛ*_oil_*ɛ*_ο_*κ*)^1/2^=(8*γa*/*ɛ*_oil_*ɛ*_ο_*κ*)^1/2^. Given that *κ*∼1 and *ɛ*_oil_∼1, the minimum voltage required for a particle of radius *a*∼10 μm to be removed from the bulk liquid interface is on the order of hundreds of volts.

### Experimental realization

**Figure 2:**
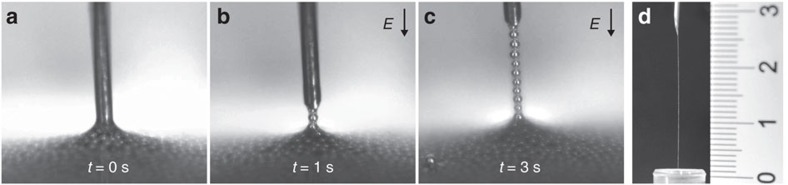
Experimental realization. (**a**–**c**) Pulling a conductive chain out of a container filled with a dispersion of Ag-coated hollow silica spheres (radius ∼30 μm, density ∼0.17 g cm^−3^) in silicone oil (viscosity=100 mPa s, density∼0.96 g cm^−3^). The electrode is first dipped into the dispersion and then an AC electric voltage (600 V, *f=*20 kHz) is applied (*t>*0 s). When the electrode is raised, a conductive chain is pulled out of the dispersion. (**d**) A nearly 3-cm-long chain comprising hundreds of particles can be formed within a minute (ruler in centimeters). A similar experiment is presented in Supplementary Movie 1.

To realize this approach in experiment, we employ a dispersion of silver-coated hollow silica microspheres (*a*∼30 μm, *ρ*∼0.17 g cm^–3^) in silicone oil (viscosity *η*=100 mPa s, mass density *ρ*_oil_∼0.96 g cm^–3^, surface tension *γ*∼0.03 N m^–1^). After dipping the electrode into the dispersion, we apply an electric voltage of magnitude *U*=600 V between the electrode and the bottom of the container. To eliminate any unwanted DC effects, such as contact charge electrophoresis^29^, we employ a high-frequency AC field (*f=*20 kHz, square wave). The electrode is elevated at an average speed of around 1 mm s^–1^. As illustrated in Fig. 2, in less than a minute, a nearly 3-cm-long chain comprised of around 500 particles is easily formed and stays in place even after the field is turned off.

**Figure 3:**
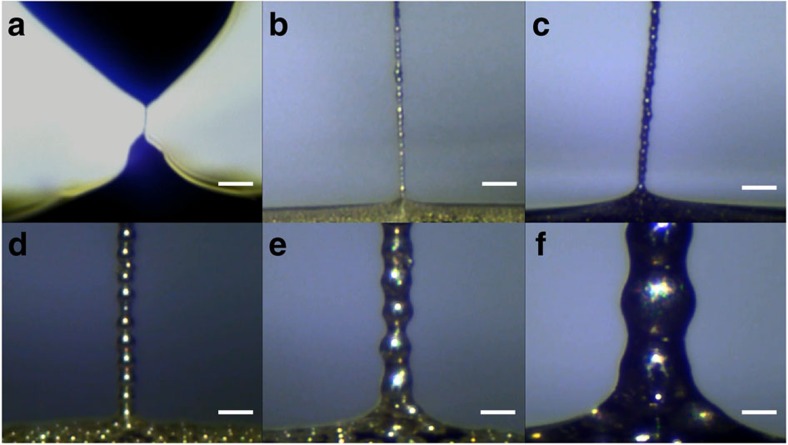
Viability for a wide range of particle sizes. (**a**) ∼100 nm; (**b**) ∼15 μm; (**c**) ∼25 μm; (**d**) ∼55 μm; (**e**) ∼100 μm; (**f**) ∼200 μm. The structure in **a** is a chain of nanoparticle aggregates rather than individual particles, with a thickness of about 1 μm. (**a**) Scale bar, 20 μm; (**b**–**f**) scale bar, 100 μm. See also Supplementary Movie 2.

Moreover, as illustrated in Fig. 3a–f, our approach works for a wide range of particle diameters, spanning from 100 nm to 200 μm. Note that the structure shown in Fig. 3a is a chain of nanoparticle aggregates rather than individual particles, with a thickness of about 1 μm.

### Requirements on the applied field

Since this experimental realization confirms the viability of the concept, we further explore the underlying mechanism. Notably, we find that voltages significantly lower than the predicted threshold are sufficient. For example, for spheres of radius 30 μm, the observed threshold is ∼100 V, far lower than *U*_min_=570 V (the value obtained using the conventional DEP model). This can be understood from a significant underestimation of the force *F*_e_ in the conventional DEP model when *d*→2*a*, which arises from omission of the influence of the polarized sphere on the electric field. In reality, when two spheres are nearly touching (surface separation *s*→0), the electric field is strongly altered, reaching a magnitude of the order of *U*/*s*, far exceeding the assumption *aU*/*d*^2^. Consequently, the induced surface charges heavily concentrate at the facing poles of both spheres, rendering the conventional DEP model invalid. To account for this we perform a finite-element analysis (FEA, see Methods), in which we apply a voltage *U* on one sphere and obtain the DEP force on the other by computing the induced surface charges and directly summing the electrostatic forces exerted on them.

**Figure 4:**
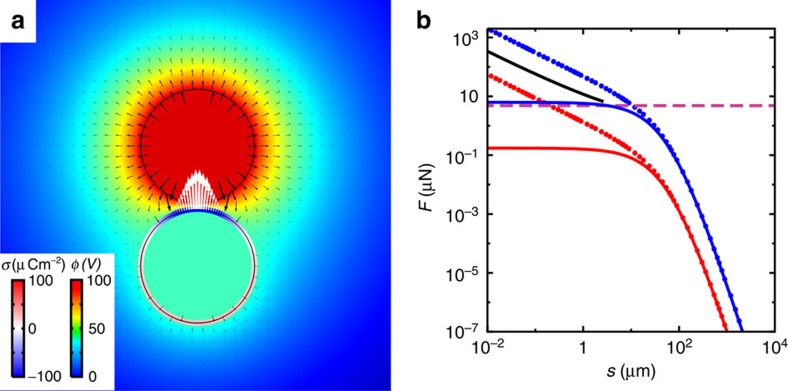
DEP forces. (**a**) Electric field generated by a spherical electrode at *U*=100 V and a polarized metal sphere right beneath it. The two spheres (solid circles) have radius *a*=30 μm and are separated by *s*=3 μm. Other details are provided in the Methods section. The induced surface charge density *σ* (on the lower sphere) and the electric potential *φ* (throughout space) are shown as colour maps. Electric field *E* and resultant surface forces *f*=*σE* are presented as black and white arrows, respectively. (**b**) Dependence of DEP force on particle surface separation *s*. Two different voltages are considered, *U*=100 V (red) and *U*=600 V (blue), respectively. For each voltage, we compare the result from the finite-element analysis (FEA, dots) and that from the conventional DEP model (solid curves), that is, *F*_e_=2π*ɛ*_oil_*ɛ*_o_*a*^3^*κ*∇(*E*^2^)=8π*ɛ*_oil_*ɛ*_o_*a*^5^*κU*^2^/(2*a*+*s*)^5^. The functional form of the asymptotic solution *F*_e_∝1/[*s*(ln *s*)^2^] as *s*→0 is plotted as the black curve. The capillary force *F*_sp_≈5.6 μN is also shown (dashed line).

As illustrated in Fig. 4, the electric field generated by the sphere at voltage *U* is strongly distorted by the nearby polarized sphere. Indeed, at small separations *s*, the resultant DEP force follows the exact asymptotic solution *F*_e_∝1/[*s*(ln *s*)^2^] (refs 30, 31), exceeding the conventional DEP model by more than an order of magnitude when *s*<0.1*a*. This explains the experimental observation that voltages as low as ∼100 V are sufficient.

Another subtle point is that once a conductive sphere touches the electrode, it is charged and subsequently repelled by the electrode via contact-charge electrophoresis (CCEP) (ref. 29). Supplementary Movie 3 demonstrates the standard CCEP oscillation that occurs at low AC frequency, a phenomenon potentially destabilizing the formed chain. This problem can be resolved by utilizing high-frequency AC fields (>100 Hz), which quickly desynchronize the charges on the sphere and the electrode and thus largely suppress CCEP.

### Shape control

**Figure 5:**
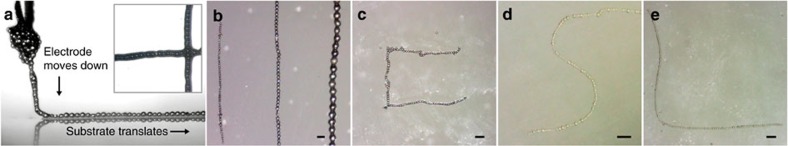
Particle deposition and 2D writing. (**a**) Direct deposition of a formed chain at a speed of approximately 0.2 mm s^−1^. Inset: Top view of a pattern formed by subsequent deposition of two individual chains. Particle size is of ∼55 μm. (**b**) Example of parallel deposition of chains composed of particles with different sizes, of ∼25, ∼55 and ∼100 μm, from left to right. (**c**–**e**) Examples of C-shaped, S-shaped and L-shaped pathways, composed of particles with sizes of ∼25, ∼15 and ∼15 μm, respectively. Scale bar, 100 μm.

The chains formed via this route remain stable even after the external field is turned off, held together by the sphere–sphere liquid bridges, which at the same time render them flexible enough to bend^32^ (Supplementary Fig. 1). This combination of properties makes it possible to subsequently deposit the formed chains into any targeted patterns via direct writing. As a demonstration, Fig. 5 presents C-shaped, S-shaped and L-shaped pathways. Such two-dimensional (2D) writing offers the potential for designing simple granular electronic circuits on an arbitrary surface without the need for prefunctionalization of the particles.

**Figure 6:**
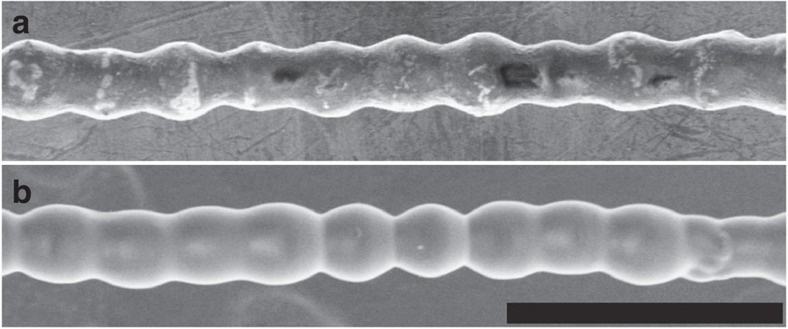
Solidification of a formed chain. Scanning-electron-microscopy images of a particle chain embedded in solidified paraffin wax (**a**) and in solidified resin (**b**). Scale bar, 200 μm.

The chains can also be easily and rapidly solidified to fix their shape. Solidification can be induced through cooling (for example, by employing premelted paraffin wax) or chemical curing (for example, with temperature- or UV-sensitive epoxies). We demonstrate two examples of solidified chains composed of conductive microparticles initially dispersed in liquid resin and in liquid paraffin wax, respectively. The resin, blended with a volatile organosilicon compound (cyclopentasiloxane), hardens as the organosilicon evaporates, which takes several minutes after chain formation. By contrast, the solidification of chains pulled out from paraffin wax preheated to around 80 °C takes only several seconds, the time needed for cooling the liquid paraffin below its melting temperature (65 °C). Figure 6 shows scanning-electron-microscopy images of a chain enveloped by solidified paraffin wax and a chain covered by solidified resin.

**Figure 7:**
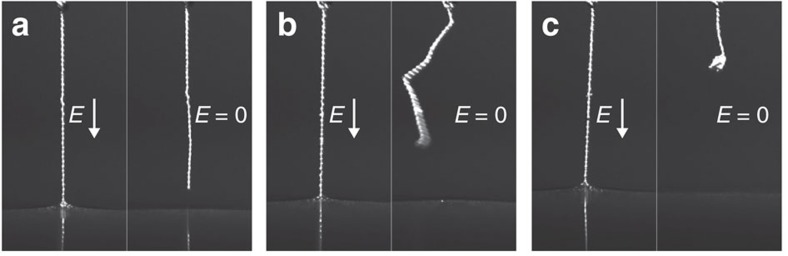
Kinking and curling up of a chain. Three pearl-necklace chains composed of particles with *a*≈30 μm and formed in silicone oil (viscosity *η*=350 mPa s) at pulling speeds (**a**) 0.01, (**b**) 0.1 and (**c**) 1 mm s^−1^, respectively. (**a**) At low pulling speed, the chain remains stable after the field is turned off. At higher pulling speeds, it kinks (**b**) and curls up (**c**). Generally, the amount of excess liquid dragged with the chain depends on the pulling speed.

One final note on the fabricating process is that the pulling speed cannot be too fast. As illustrated in Fig. 7, a chain becomes unstable in the absence of external fields when it has been pulled out too quickly, tending to kink or curl up. This instability is caused by excess liquid in the sphere–sphere bridge. The amount of liquid (per particle) dragged during pulling typically increases with pulling speed and should not exceed the maximum volume of a sphere–sphere bridge, that is, half the volume of a sphere.

## Discussion

In summary, we have presented a convenient route for fabricating one-dimensional conductive micron-sized structures, combining field-induced assembly and capillary effects. The assembled structure remains stable outside a liquid environment and even without continuous energy supply, making this method different from most conventional approaches. Since the particles are connected through liquid bridges, the chain exhibits a certain flexibility and can be utilized in 2D direct-writing processes. Solidification then permits fixation of the chain in such a designed shape. The entire process takes less than a minute for generating a long chain composed of thousands of particles. We have demonstrated that this approach works for a wide range of particle sizes and can be scaled up (Supplementary Fig. 2).

## Methods

### Experimental set-up

The experimental set-up consisted of a signal generator (SDG1025 Siglent), a high-voltage bipolar amplifier (10HVA24-BP1 HVP), *x*–*y*–*z* motorized translation stage (MTS25-Z8 Thorlabs), a digital microscope (AM7115 Dino-Lite) and a PC for collecting images. The signal electrode for pulling out the particles from a dispersion was made of a thin aluminium wire attached to another motorized translation stage for controlling the pulling rate.

### Liquids

Silicone oils (Dow Corning 200 with kinematic viscosity ∼100 cSt; electric conductivity <5 pS m^–1^; relative permittivity ∼2.5), epoxy resin (Dow Corning 749 fluid) and paraffin wax (Sigma-Aldrich ASTM D 87, melting point ∼65 °C) were used to form a dispersion with conductive particles or microspheres.

### Particles

Conductive silver-coated hollow glass microspheres (M-40-0.67/10–20 μm, M-60–0.17/53–63 μm, specific densities of 0.67 and 0.17 g cm^–3^, average size 15 and 55 μm, respectively), conductive silver-coated solid-glass microspheres (SLGMS-AG-2.5 86–110 μm, specific density 2.5 g cm^–3^, average size 100 μm), conductive stainless steel microspheres (SSMMS-7.7 23–28 μm, SSMMS-7.8 95–105 μm, SSMMS-7.8 190–220 μm, SSMMS-7.8 480–520 μm, specific density 7.8 g cm^–3^, and average size 25, 100 and 200 μm, respectively) were purchased from Cospheric LLC. Magnetic nanoparticles (637106-250G, 50–100 nm) were purchased from Sigma-Aldrich. The nanoparticles were dispersed in silicone oil using ultrasonication. But since they were not stabilized (for example, by surfactants), they formed aggregates with sizes ranging from hundreds of nanometres to a few micrometres. Thus, when pulled out from a dispersion, they formed a chain of particle aggregates rather than a chain of individual particles.

### Finite-element analysis

FEA was performed using COMSOL (Version 5.1, 2015) to investigate the DEP forces between two adjacent spheres. Two spheres of radius *a*=30 μm with dielectric constant *ɛ*_p_=10^6^, separated by distance *s*, were placed at the centre of a cylinder of radius 6 mm and height 12 mm with dielectric constant *ɛ*_oil_=2.5. The axial symmetry made it possible to reduce this 3D problem to 2D for computational efficiency. The reduced system was divided into more than 20,000 triangular elements. One sphere was kept at a voltage *U*, the other isolated, while satisfying net charge neutrality. The boundaries of the cylinder were grounded. The DEP forces between the two spheres were obtained by computing the induced surface charges and directly summing over the electrostatic forces exerted on them.

### Data availability

The data that support the findings of this study are available from the corresponding authors upon request.

## Additional information

**How to cite this article:** Rozynek, Z. *et al*. Formation of printable granular and colloidal chains through capillary effects and dielectrophoresis. *Nat. Commun.* **8,** 15255 doi: 10.1038/ncomms15255 (2017).

**Publisher's note**: Springer Nature remains neutral with regard to jurisdictional claims in published maps and institutional affiliations.

## Supplementary Material

Supplementary Information Supplementary Figures.

Supplementary Movie 1 Pulling a conductive chain out of a dispersion of silver coated hollow silica spheres (radius ~30 μm) in silicone oil (η= 50 mPa·s). The electrode is elevated at an average speed of around 1 mm·s^-1^.

Supplementary Movie 2 Viability over a wide range of particle size: (a) ~100 nm, (b) ~15 μm, (c) ~25 μm, (d) ~55 μm, (e) ~100 μm, (f) ~200 μm. The structure in panel (a) is a chain of nanoparticle aggregates rather than individual particles, with thickness of about 1 μm.

Supplementary Movie 3 Contact charge electrophoretic particle oscillations. A granular chain composed of 100 μm stainless steel particles is assembled in silicone oil, and then the electric voltage is turned off to enable fracturing of the chain. Once the voltage is turned on again, the fractured chain reconnects. At high frequencies of the electric voltage (above ~100 Hz), the entire chain moves back towards the electrode. For lower frequencies, particles may experience an oscillating motion due to the contact charge electrophoresis. This effect is demonstrated here at frequencies 1 Hz and 1 mHz.

## Figures and Tables

**Figure 1:**
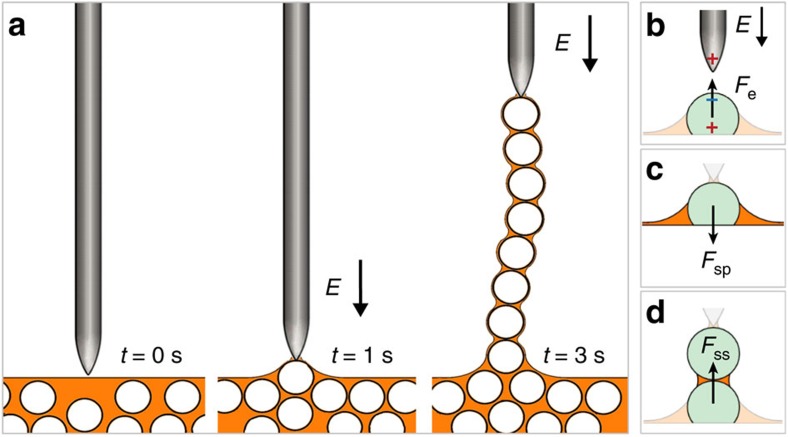
Schematics of chain formation. (**a**) Particles are pulled out of a dispersion to form a ‘pearl necklace', by applying an electric field through a needle-shaped electrode. (**b**,**c**) A particle at the air–liquid interface experiences an upward dielectrophoretic force *F*_e_ and a downward capillary force *F*_sp_ stemming from the sphere–plane liquid bridge. (**d**) Once the particle is pulled out, it automatically forms a sphere–sphere bridge with the particle below it, and pulls that particle upwards with force *F*_ss_.
