# Supplementary material for: Formation of printable granular and colloidal chains through capillary effects and dielectrophoresis
Source: Nat Commun. 2017 May 12;8:15255. doi: 10.1038/ncomms15255 (PMC5437296; doi:10.1038/ncomms15255)
Supplement: Supplementary Information — Supplementary Figures. [file ncomms15255-s1.pdf]

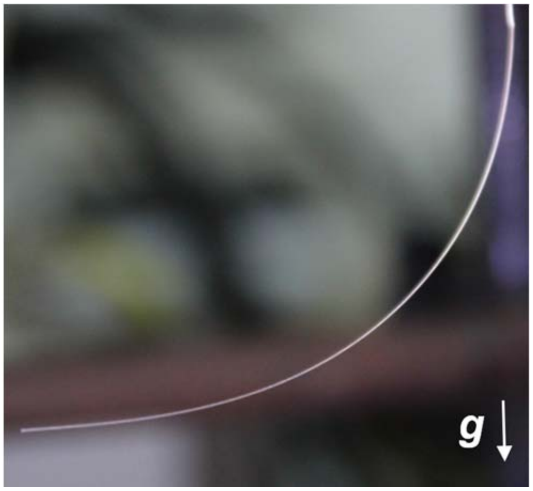

Supplementary Figure 1. **Chain flexibility.** Sphere–sphere capillary liquid bridges lend flexibility to the pearl necklace chains. Here, the chain bends towards the grounded electrode placed at the bottom-left corner of the image.

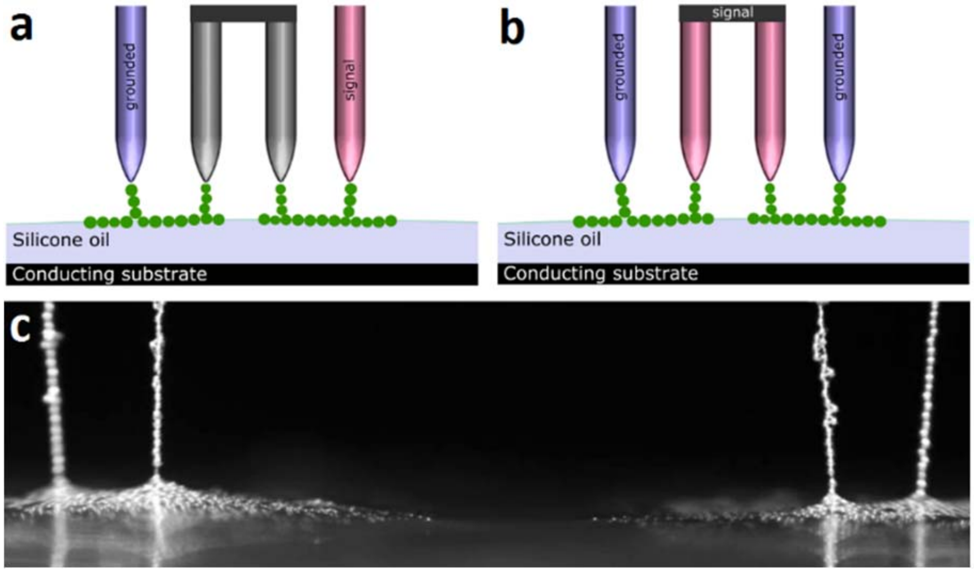

Supplementary Figure 2. **Multiple chain formation.** Schematic set-up for multiple chain formation in series (**a**) or in parallel (**b**). (**c**) Experimental realization of simultaneous formation of four chains using a parallel configuration.
